# A Mini-Thoracotomy Retropleural Microdiscectomy for Thoracic Disc Herniation: A Case Report

**DOI:** 10.7759/cureus.94142

**Published:** 2025-10-08

**Authors:** Geok Hwee Teo, Mohd Hisam Muhamad Ariffin, Suffian Sabri

**Affiliations:** 1 Orthopaedic Surgery, Melaka Hospital, Melaka, MYS; 2 Spine Surgery, Universiti Kebangsaan Malaysia Medical Centre, Kuala Lumpur, MYS

**Keywords:** micro-discectomy, mini thoracotomy, thoracic disc herniation, thoracic myelopathy, transthoracic retropleural approach

## Abstract

Symptomatic thoracic disc herniation (TDH) is relatively rare, most commonly occurring below the T8 level due to increased spinal mobility. A small herniation could cause significant spinal cord compression due to the narrow thoracic spinal canal. A 62-year-old Malay man presented with thoracic myelopathy and was unable to walk. Magnetic resonance imaging (MRI) demonstrated a large paracentral disc herniation at T9/T10 compressing the spinal cord. He underwent a lateral mini-thoracotomy retropleural microdiscectomy. Both lungs were ventilated throughout the procedure. A lateral mini-open incision measuring approximately 4-5 cm was made, and the underlying rib was segmentally resected. An exoscope was used to enhance visualization. Retropleural dissection was performed while keeping the pleura intact. Using a matchstick burr, a 1.5 × 1.5 cm box-shaped cavity was created in the posterior vertebral bodies. This cavity facilitated microdiscectomy by providing space for decompression and visualization of the ventral aspect of the spinal cord without manipulation. Intraoperatively, the herniated disc was hard and adherent to the dura, appearing chronic in nature, but was successfully removed. Postoperatively, his neurological function improved, and he regained the ability to walk. TDH is uncommon and often diagnosed late due to subtle initial symptoms and signs. Surgical decompression is indicated for patients with progressive neurological deficits. Anterior approaches allow direct access to ventral pathology without spinal cord manipulation. Mini-thoracotomy retropleural microdiscectomy offers significant advantages over open thoracotomy, including reduced blood loss, postoperative pain, pulmonary complications, hospital stay, and overall morbidity. In conclusion, a lateral mini-thoracotomy retropleural microdiscectomy offers a safe, less invasive yet effective approach to decompress the spinal cord while minimizing perioperative morbidity.

## Introduction

Symptomatic thoracic disc herniation (TDH) is relatively rare, accounting for only 0.25-0.75% of all herniated discs [1]. Unlike the cervical and lumbar regions, the thoracic spine is relatively rigid due to its attachment to the rib cage. However, the rigidity decreases caudally due to false and floating ribs, which are not directly attached to the sternum. As a result, the majority of TDH cases occur below the T8 level [1-2]. The thoracic spinal canal is narrower compared to other regions of the spine. Even a small herniation can exert significant pressure on the spinal cord and lead to neurological deficits. Patients often present with progressive thoracic myelopathy, which, if untreated, could result in paraplegia and significantly impair the quality of life [3]. Surgical treatment is usually required in symptomatic cases and has evolved significantly with advances in technology. Classical posterior approaches often result in inadequate decompression of the spinal cord. In this report, we present a case of TDH at the lower thoracic level causing myelopathy and paraplegia, which was successfully managed with a mini-thoracotomy retropleural microdiscectomy.

## Case presentation

**Figure 1:**
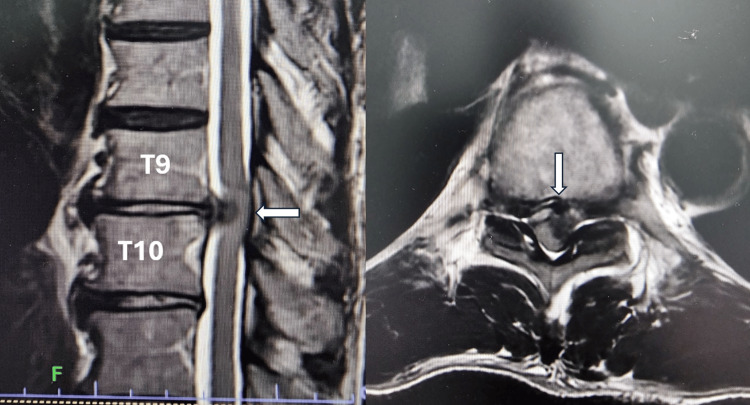
MRI demonstrated a large paracentral disc at T9/T10 compressing the spinal cord.

A 62-year-old Malay man presented with a 2-week history of back pain radiating to the epigastric region. He subsequently developed bilateral lower limb weakness and was unable to ambulate. There was no urinary or bowel incontinence. Examination revealed upper motor neuron signs in bilateral lower limbs, including hypertonia and brisk reflexes. Muscle power in his lower limbs was grade 3 (Medical Research Council grading), with sensory deficits from T11 downward. Magnetic resonance imaging (MRI) demonstrated a large paracentral disc herniation at T9/T10, compressing the spinal cord (Figure 1). He underwent a mini-thoracotomy and microdiscectomy. Intraoperatively, the herniated disc was hard and adherent to the dura, appearing chronic in nature, but was successfully removed. Postoperatively, the patient’s radicular pain resolved. Within a week, he regained full lower limb muscle power and was able to ambulate independently with a walker.

Surgical technique

**Figure 2:**
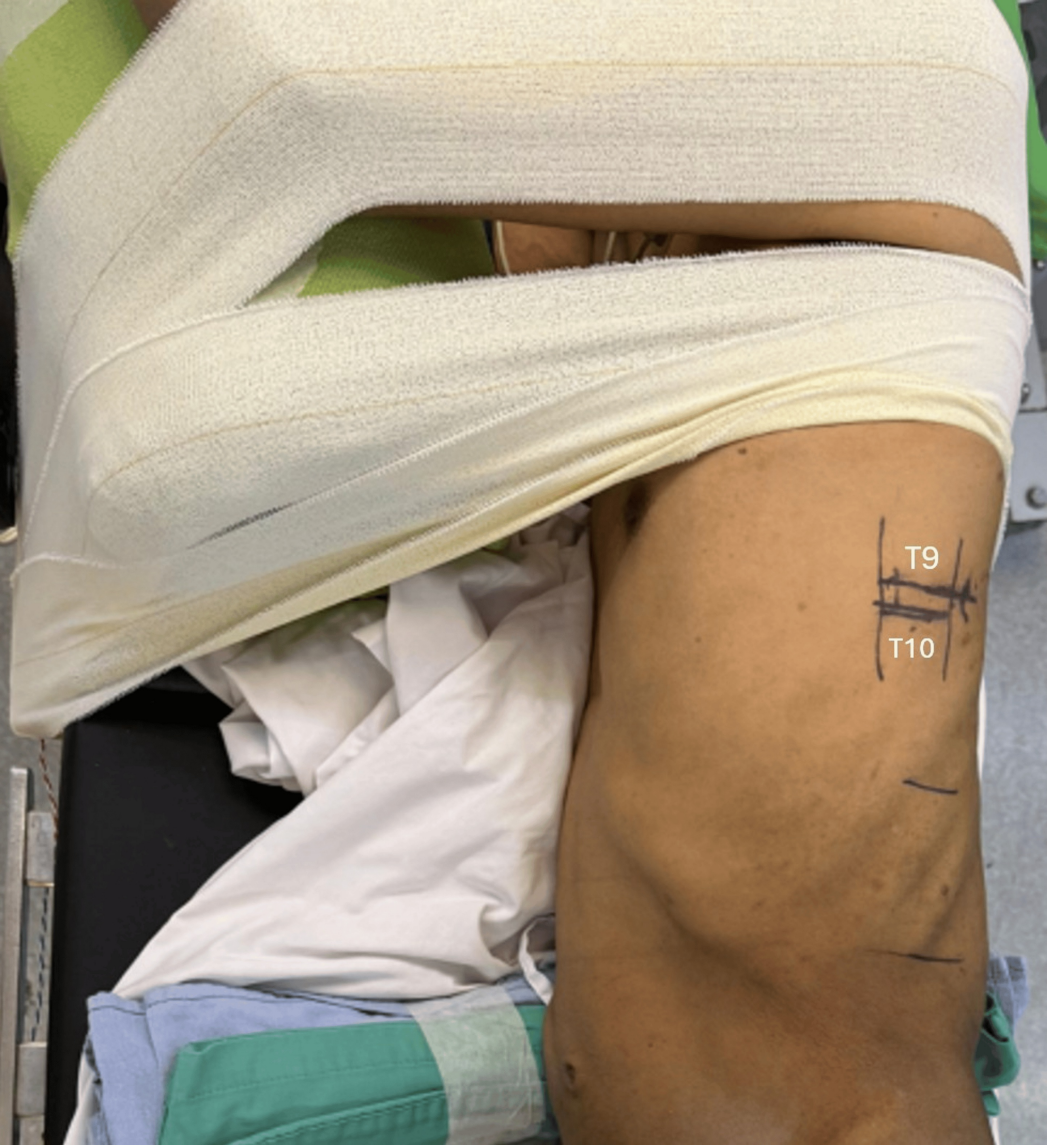
Right lateral decubitus position. T9/T10 disc and adjacent vertebral bodies were outlined.

Proper positioning of the patient prior to surgery is crucial. The patient was placed in the right lateral decubitus position under general anesthesia. The position was secured to the operating table using adhesive tape to avoid movement during the surgery. All bony prominences were well-padded to prevent pressure sores. Intraoperative neuromonitoring was employed. Using a C-arm image intensifier, the level of interest was identified. The T9/T10 intervertebral disc and adjacent vertebral bodies were outlined (Figure 2). Both lungs were kept ventilated throughout the surgery.

**Figure 3:**
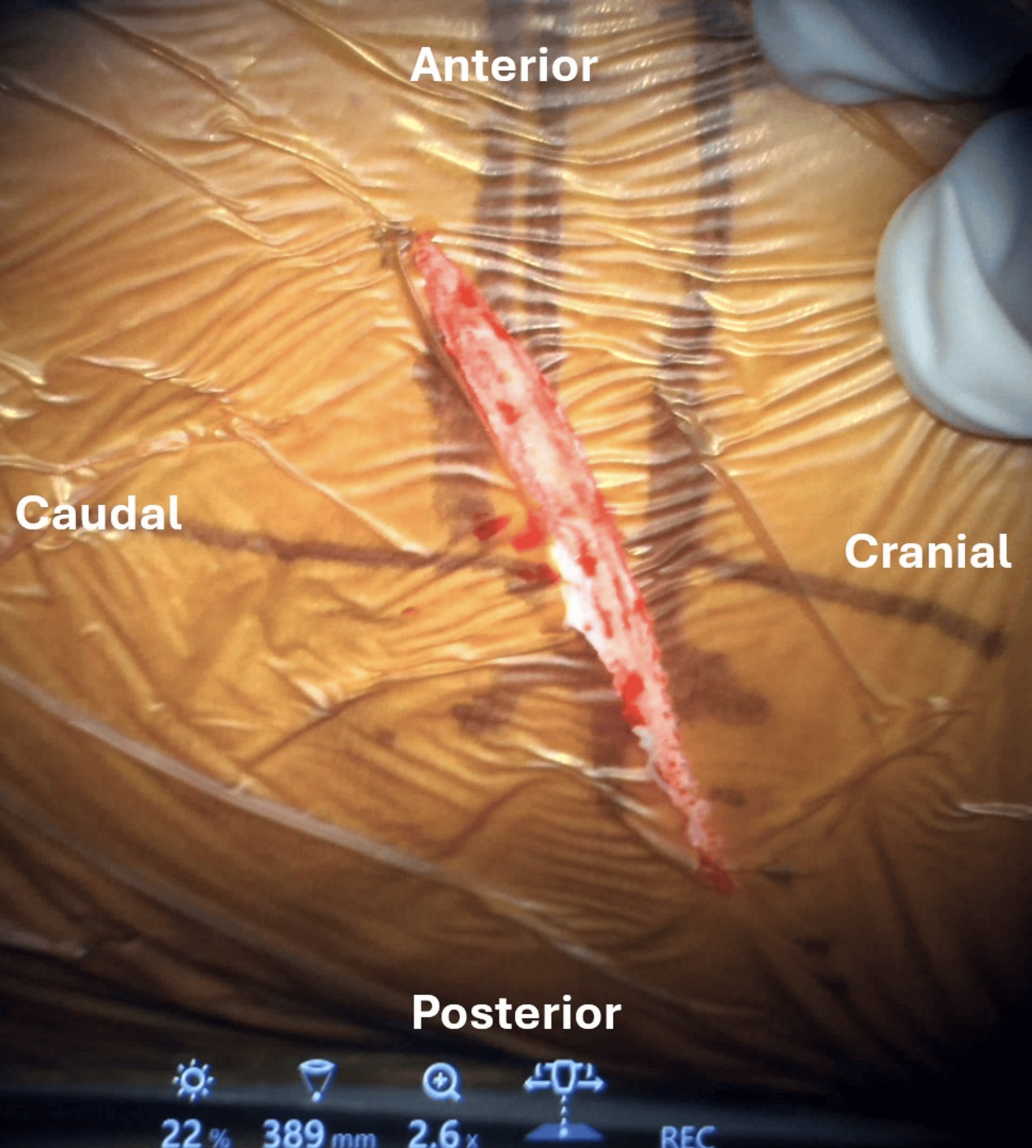
A skin incision approximately 4-5 cm parallel to the rib.

An incision approximately 4-5 cm in length was made parallel to the rib (Figure 3). The underlying rib was exposed subperiosteally, and the underlying parietal pleura was detached, with care taken to avoid injury to the neurovascular bundles running along the inferior border of the rib. The rib was then resected segmentally and preserved for later use as a bone graft. The parietal pleura was further detached from the posterior chest wall to expose the rib head and lateral vertebral body. Oblique lateral interbody fusion (Medtronic OLIF51) retractors were inserted carefully to avoid breaching the pleural cavity. A chest tube is not required postoperatively if the parietal pleura remains intact throughout the procedure.

**Figure 4:**
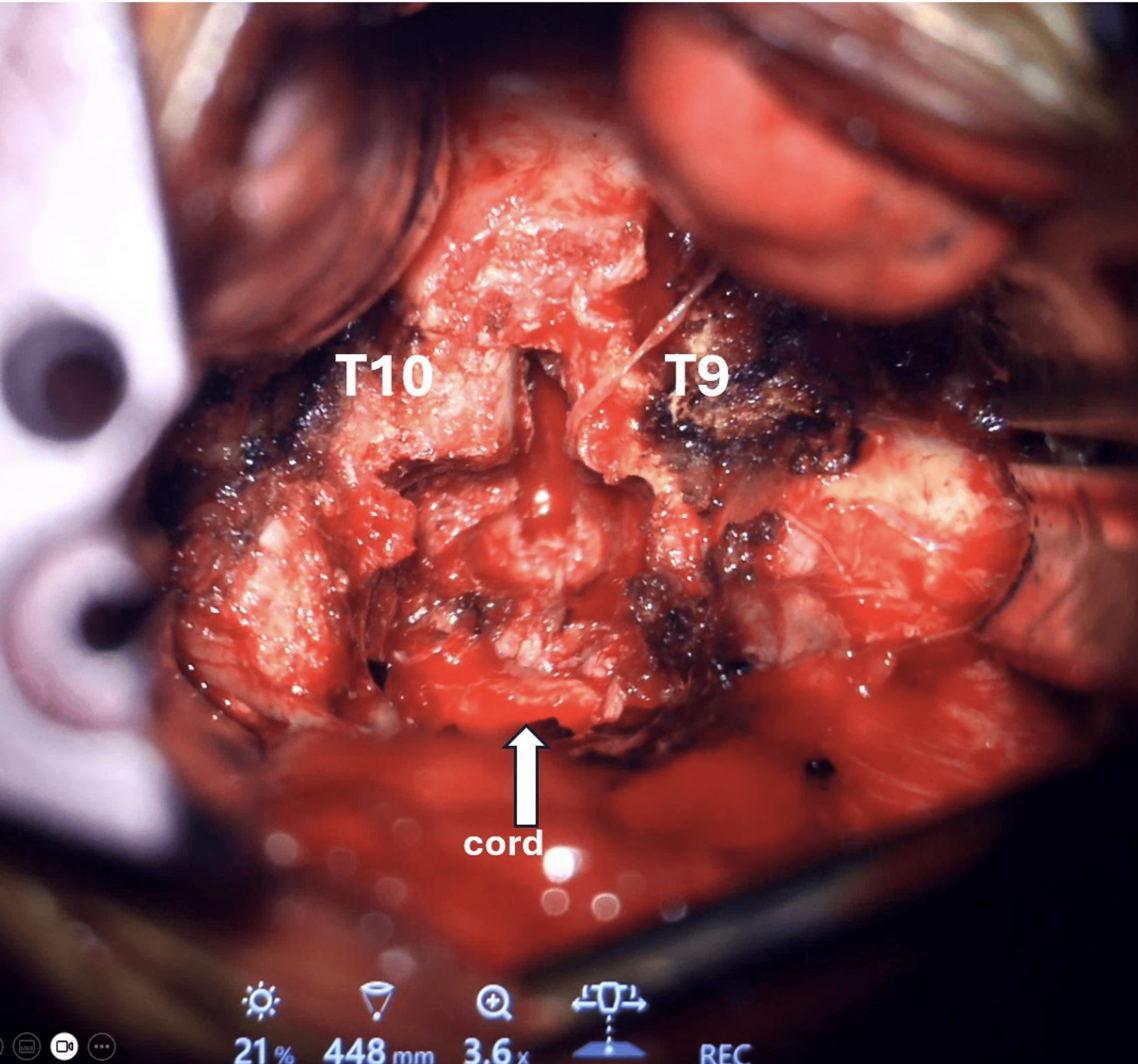
A box-shaped space was created to facilitate decompression.

The surgical level was reconfirmed using the C-arm. An exoscope was used to magnify the operating field. The rib head was removed to expose the pedicle and neuroforamen. Using a matchstick burr, a 1.5 × 1.5 cm box-shaped cavity was created in the posterior vertebral bodies (Figure 4). This cavity facilitated the microdiscectomy by creating a space for decompression and visualization of the ventral aspect of the cord without the need to manipulate it.

**Figure 5:**
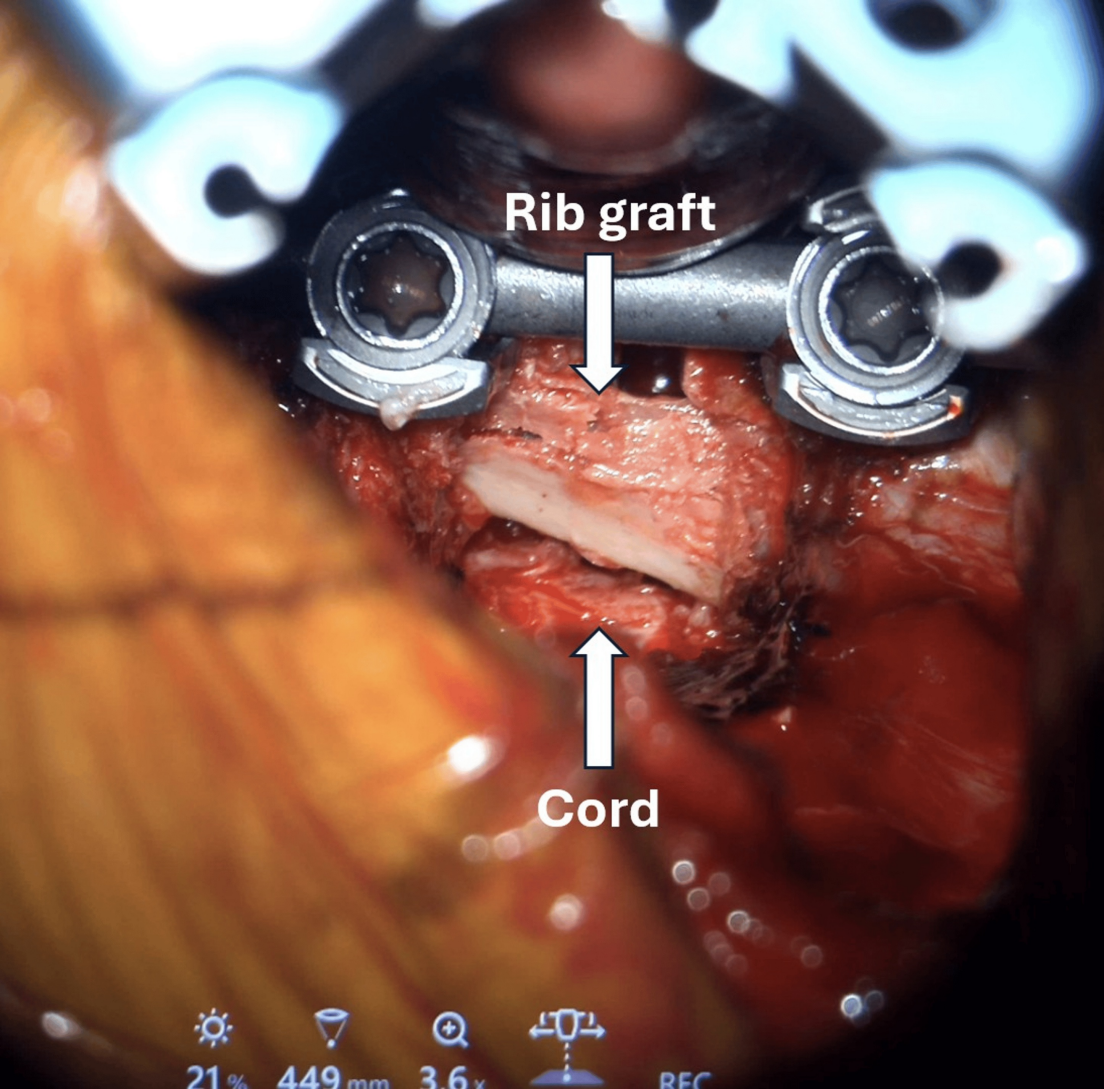
The cavity was filled with an autograft harvested from the resected rib. The spine was stabilized with pedicle screws and a rod.

**Figure 6:**
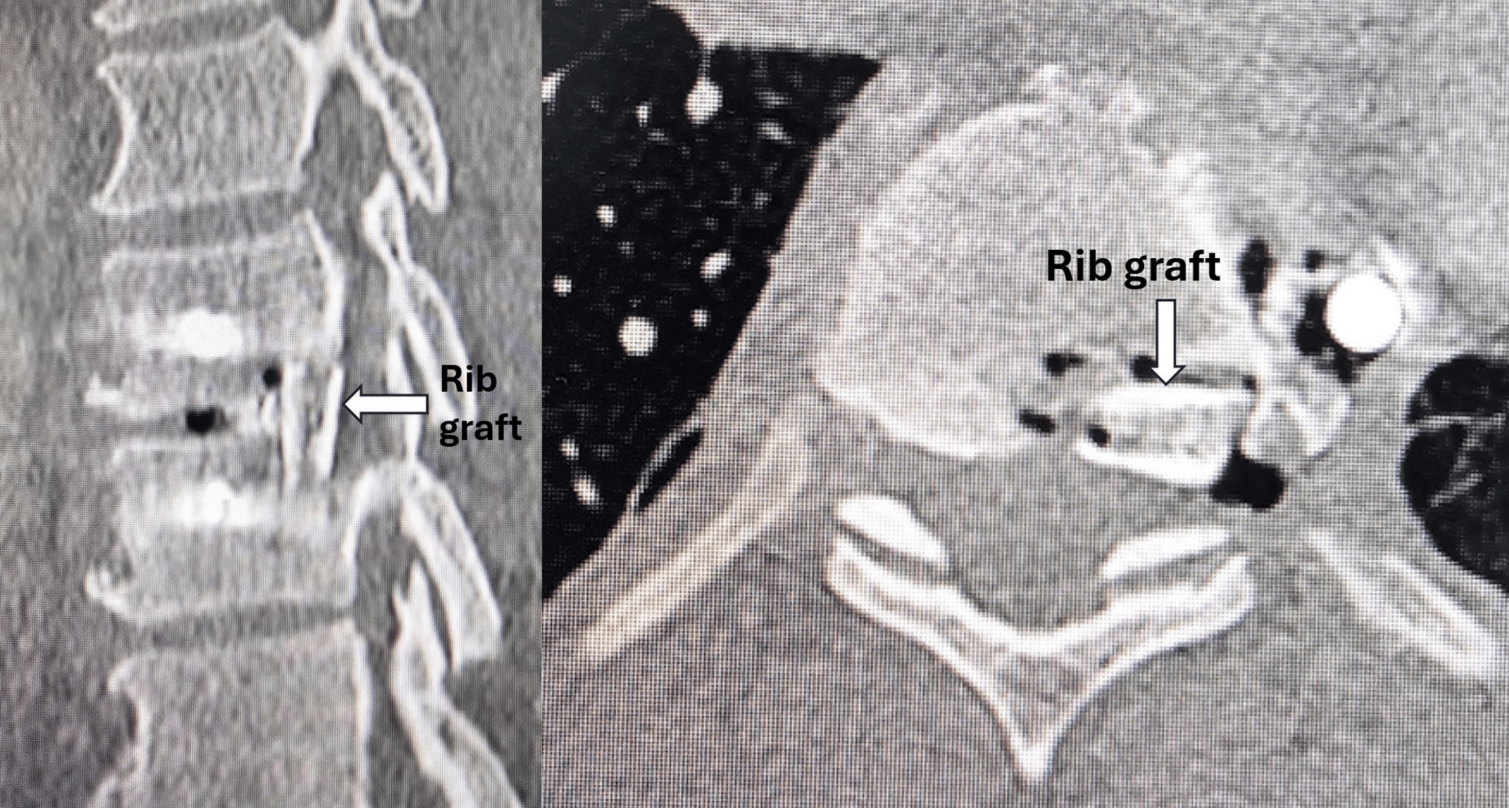
Postoperative CT scan demonstrated that the rib graft filled the void.

**Figure 7:**
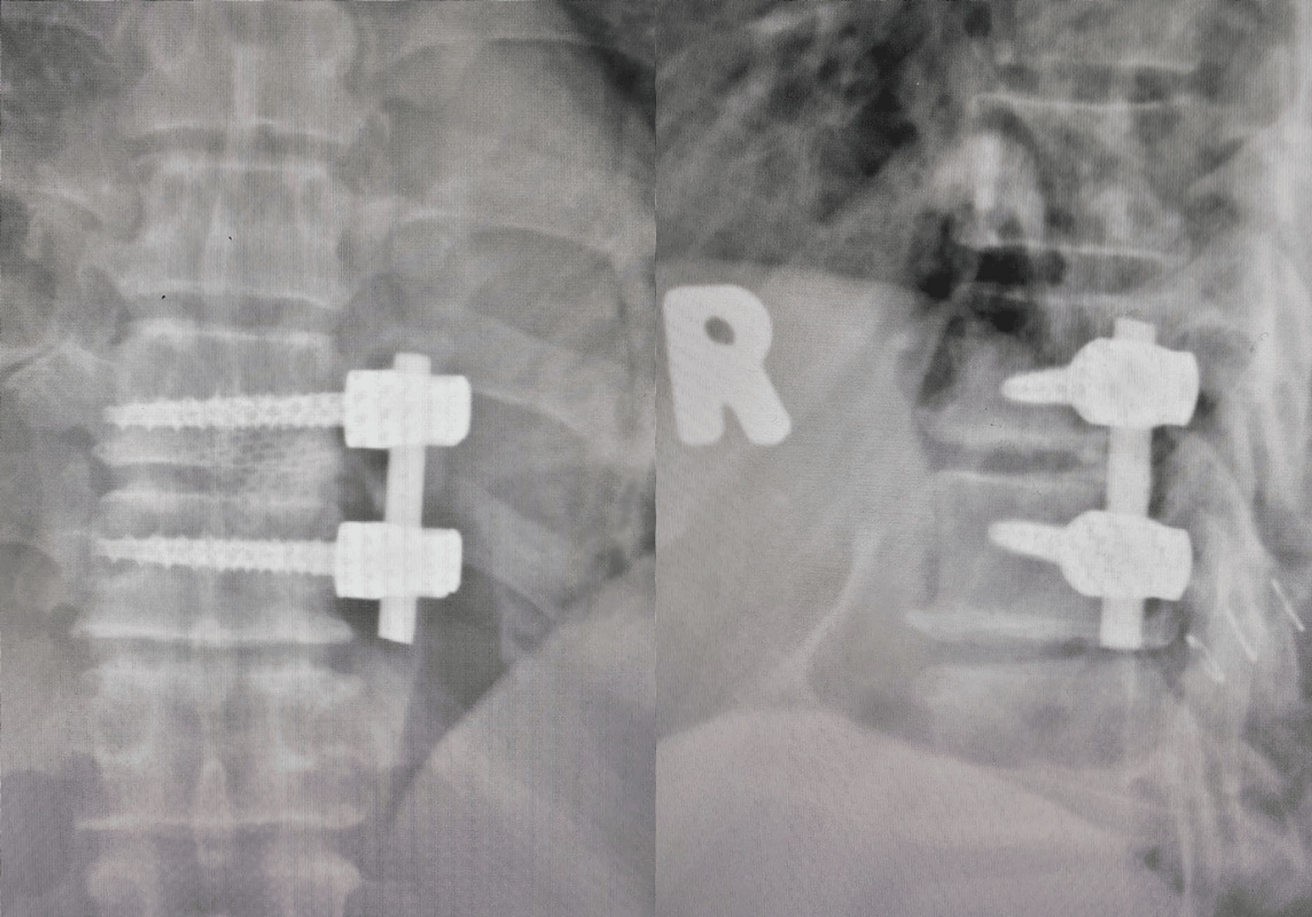
Postoperative plain radiograph showed the implant in situ.

The herniated disc, which was hard and adherent to the dura mater, was successfully removed. The cavity was filled with autograft harvested from the resected rib and shaped into sticks. Pedicle screws and a rod were inserted to achieve fusion at T9-T10 (Figure 5). A postoperative CT scan demonstrated that the rib graft filled the void (Figure 6), and the plain radiograph showed that the implant was in situ (Figure 7).

## Discussion

TDH is uncommon and often diagnosed late due to subtle early symptoms and signs. Diagnosis is usually made only after patients develop significant lower limb weakness and myelopathy. Surgical decompression is indicated for progressive neurological deficits. While posterior approaches historically dominated, they risk inadequate decompression due to limited ventral access, often resulting in poor neurological recovery [4]. Conversely, anterior approaches directly address ventral pathology without manipulating the spinal cord.

Baram et al. retrospectively analyzed 54 patients: 34 underwent anterior decompression and 20 underwent posterior decompression [5]. They found that the anterior approach group had greater neurological recovery.

Soda et al. retrospectively reviewed 94 cases comparing transpleural and retropleural approaches and found the transthoracic group had higher rates of pulmonary complications and postoperative neuralgia [6]. They concluded that the minimally invasive retropleural approach is a safe and effective technique with lower morbidity compared to the transpleural approach.

Mini-thoracotomy approaches offer significant advantages over open thoracotomy, including reduced blood loss, postoperative pain, pulmonary complications, hospital stay, and overall morbidity [7-9]. In our practice, the mini-thoracotomy retropleural approach allows direct access to the anterior pathology without violating the pleural cavity.

Alan et al. studied 30 patients who underwent a mini-open lateral retropleural approach and generally did not require a chest tube [10]. They suggested that a chest tube is indicated only when the pleural cavity is violated intraoperatively and a pneumothorax or hemothorax ≥3 cm is evident on postoperative chest radiograph.

With advancements in technology, conventional open thoracotomy is no longer required for TDH. We used an exoscope to enhance visualization. The exoscope provided excellent detail of the operating field and enabled precise and safe decompression. The mini-thoracotomy technique is associated with favorable outcomes, including reduced morbidity and faster recovery [11]. Our patient’s excellent neurological recovery highlights the efficacy of this approach.

## Conclusions

TDH presenting with progressive neurological deficits is uncommon but clinically significant. In view of its rarity, a high index of suspicion is essential for early diagnosis. Prompt recognition and timely surgical intervention are crucial in preventing irreversible neurological compromise. Among the various surgical options, a lateral mini-thoracotomy retropleural microdiscectomy offers a safe, less invasive yet effective approach to decompress the spinal cord while minimizing perioperative morbidity.
